# Supplementary material for: Tandem Gene Clusters as Phylogenetic Anchors Reveal the Hidden History of Vertebrate Visual Opsins
Source: Mol Biol Evol. 2025 Oct 1;42(10):msaf231. doi: 10.1093/molbev/msaf231 (PMC12486383; doi:10.1093/molbev/msaf231)
Supplement: msaf231_Supplementary_Data [file msaf231_supplementary_data.zip › Additional file 1.pdf]

Additional file 1: Short-headed lamprey rhodopsin (*RH1*) coding sequence

```
>c117566_g1_i1 Mordacia mordax RNA-seq assembly v1 len=2047 path=[1:0-83 85:84-84
86:85-504 5479:505-506 5482:507-1201 @1203@!:1202-1635 4843:1636-1639 1637:1640-
1746 1744:1747-2046]
CCGCCAATTAGACCCTGTGCTGCCACACAGCCACAAAGCTACCCCTAAGCCGACCCTCCAACCCTCCCTCCACGGGCGGG
GAGAGGGGGGGGGATTCCCCAGATGTCGCCCGGATCTTCTGGTTGCCGGTCCCAGGTTCCTCAAGCCGGGGAGACCTGGC
TGGTGGACACCGAGGACACCTCCGTCTTGCTCGTGGAGGCTCCCGAGTCATCGTCGCCCAGAGGATTCTTGCCGCAGCAC
AGCGTGGTGATCATGCAGTTGCGGAACTGCTTGTTCATGAGGATGTAGATGACCGGGTTGTAGAGGGACGAGCTCTTGGC
AAAGAAGGCCGGCGCGGTCATGAAGGTGGCGCCGAAGTCGGACCCCTGGTTGGTGAAGATGTAGAACGCCACGGAGGCGT
AGGGCACCCAACACACCAGGAATCCCACCACCATCAGTACCACCATGCGGGTCACCTCTTTCTCGGCCTTCTGCGTCGAC
GCCGACTCTTGCTGGGCCGCGGCCGCCTCCTTGACTGTGCAGAGCAAGCGTCCGTAGGAGAAGAAAATGATCACGAAAGG
GATGACGAAGTGTACGACGAACATGTAGATGACGAACGACTCGTTGTTGAAGTCGGGGTTCATCGTGTAGTAATCCGGCC
CGCACGAGCATTGCATTCCTTCCGGAATGAACCTGGACCAGCCTAGGAGTGGCGGAGCAGCGCATGACAAGGCCATGATC
CACGTGAAAGCCACCCCCATGATGGCGTGGGTGCTGCCGAAGCGGAAGTTGCCCATGGGTTTGCAAATGACGATGTAGCG
CTCAATGGCGAGCGCCACAAGGGACCAGAGAGACACTTCGCCGCCGAGCGTGGCAAAGAAGCCCTCCGTGGAGCACATGG
TGGGTCCGAAGATGAAGTAGCCGTTCATGGAGGTGTACATGGTGACGGTGAAGCCGCAGCAGATCATGAAGAGGTTGGAC
ACGGCGAGGTTGAGCAGGATGTAGTTGAGCGGGGTCCTCAGCTTCTTGTGCTGCACCGTGACGAACAGCGTGAGGAAGTT
GATGGGGAAGCCGACGAGGATGAGGAAGAACATGTAGGCAGCCAGGGCGGAGAACTTCCATGGTTCGGCCAGGTAGTACT
GCGGGTACTCGAAAGGACTGCGAACCACTCCGGTCTTGTTTGAGAACGGGACGTAGAAATTCTGTCCCTCTGTGCCGTTC
ATGGTGCTTGCTGTTGCCGAGTTCCCTGGTGCGTGCGGTGGTGAGATTTAACCAATGGGCACAAGTAGACGTGTGTGTTT
GCGATGTCTCCCGTGTGGATTGAATTCGTAAGTATTGCCTTGTTGTCGAGTGATTTGCTTAAAATGTTTTTTTTTTAAAG
TCGTTTAACGGTTCAATTGAACAATTTGTTTTGATATTGGTAGTGGGTAGTGATGATACGTGTTGTGGTTTAAAAGCGTG
ATTAGTTCCAGTCGCGCCCACTTTGCCAAGAGTCTGATCGTGCGAGAGATGGCTTTCGCGCTGATGAAATAACTATAAAG
TGTTCATCAGAATAGGCGCTCGCTAACAGCAGTCGTCCACAGCAGACTTTATGAAGGCTGATGGCTCGATTGTGTTAACG
TGAATTAATGTGTACCACGAAGACGCAAGTGGACTGTGTTTGTCATGTGTTCATGTACACATGTGATGTAGGCGCACTAA
ACTTGTGTGAACTTGACGTTAATGTAATATCTACGTGTGTCGCTAATGTCCAGTCATGTGTGCACTGCGGTGGTGTTTCA
GTTAAGTGATGATCAACGGTATCTAGAGTTCTATGCTGTGCAGACAAGCGCTCGCTCAAGCGTTCGATACGGTGCGCGAT
ATGCAACGGCCGGTCGCTGGAATATACATAAATTCTGCACGACTGTGTAATCGTGTAGCTTGGGCACGTGGCGCGTTTGC
CAGAGCGTTAAATTGGATTCGCGCGTTCGTGGGTGCAAGGAAATGCCCTTGAGTTAAGCGATGACTGATGGCAGTGGCGC
ACGCGCCGTGCGGGTGCCGTGGTGCTGGTCCGTTCAGCAAGGGGGGG
```
